# A Woman with Abdominal Pain After Lap-belt Trauma

**DOI:** 10.5811/cpcem.1251

**Published:** 2023-11-08

**Authors:** Chandler Davis, Erin F. Shufflebarger, Andrew Hubbs, Zachary S. Pacheco

**Affiliations:** * University of Alabama Birmingham Heersink School of Medicine, Birmingham, Alabama; † University of Alabama Birmingham Heersink School of Medicine, Department of Emergency Medicine, Birmingham, Alabama

**Keywords:** traumatic abdominal wall hernia, blunt abdominal trauma, handlebar hernia

## Abstract

**Case presentation:**

A 24-year-old female presented to the emergency department with diffuse abdominal pain after involvement as a restrained driver in a motor vehicle collision (MVC). Computed tomography of the abdomen revealed a traumatic abdominal wall hernia due to rectus wall rupture with complete bowel herniation.

**Discussion:**

A traumatic abdominal wall hernia is a rare complication of blunt abdominal trauma that is typically associated with injury from a motorcycle handlebar but is more commonly seen after a MVC. It is important to consider this diagnosis when evaluating patients with abdominal pain after blunt abdominal trauma from either of these mechanisms.

CPC-EM Capsule What do we already know about this clinical entity?

*A traumatic abdominal wall hernia is a rare complication of blunt abdominal trauma that is typically associated with injury from a motorcycle handlebar.*

What is the major impact of the image(s)?

*This case describes the under-reported but more common mechanism for a traumatic abdominal wall hernia following a motor vehicle collision.*

How might this improve emergency medicine practice?

*Emergency physicians should consider this diagnosis when evaluating patients with abdominal pain after blunt abdominal trauma from a motorcycle or vehicle collision.*

## CASE PRESENTATION

A 24-year-old female presented by ambulance as a trauma alert to the emergency department after a head-on motor vehicle collision (MVC). Emergency medical services reported that she was restrained with only the lap portion of the seatbelt. On arrival, the patient reported loss of consciousness in the field, abdominal pain, and bilateral hip and leg pain. The primary exam was notable for an intact airway, as well as adequate breath sounds and circulation. She was initially tachycardic with a heart rate of 133 beats per minute; her other vital signs were stable. She was afebrile, normoxic at 97% on room air with 20 respirations per minute, and her blood pressure was 128/76 millimeters of mercury.

**Image 1.**
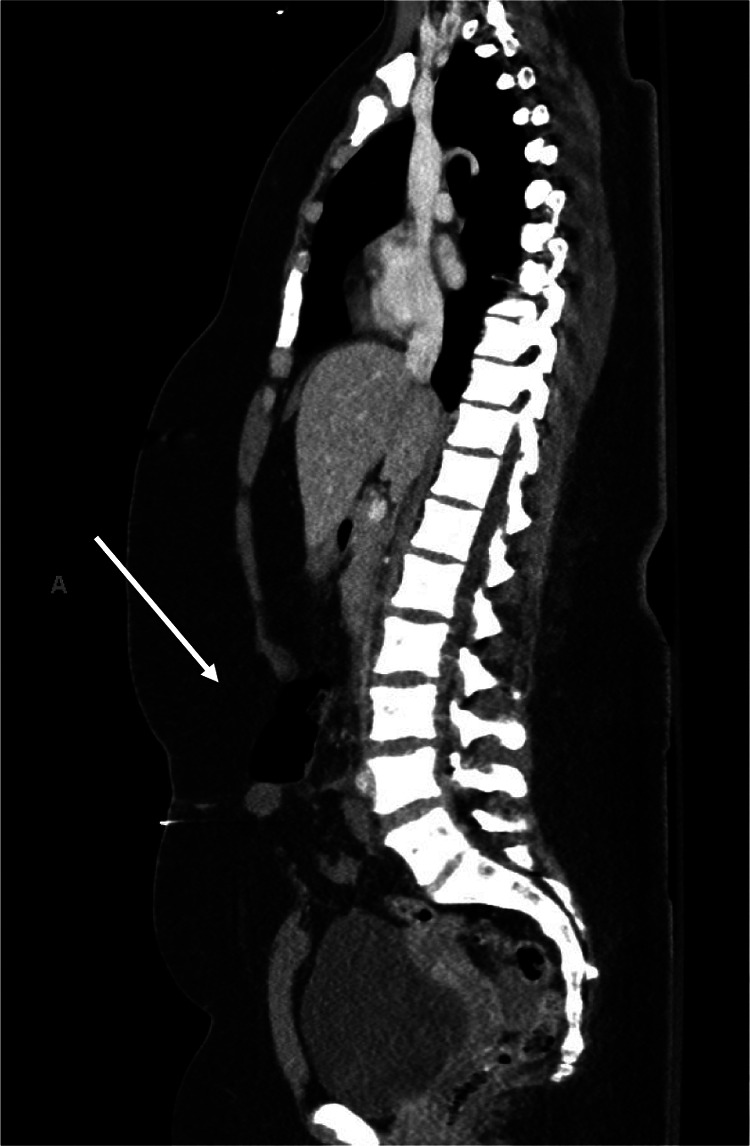
Sagittal computed tomography showing rectus muscle disruption with abdominal content herniation (arrow).

**Image 2.**
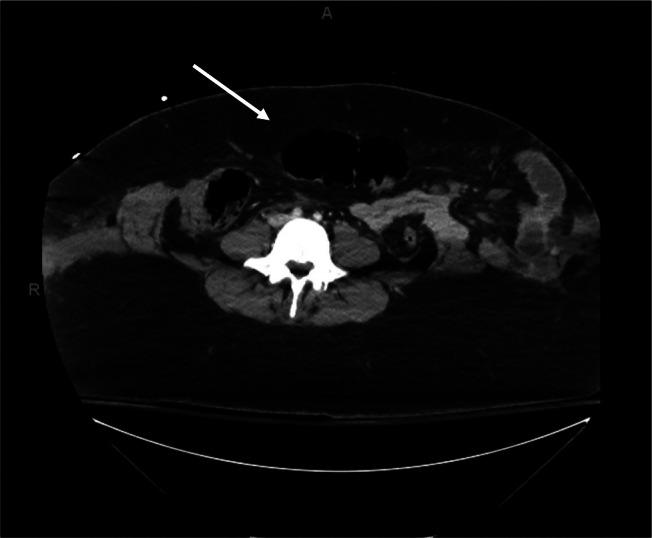
Transverse computed tomography showing large traumatic ventral hernia (arrow).

The secondary exam was notable for an immobilized cervical spine, ecchymosis across the lower abdomen, and diffuse abdominal tenderness. Labs were drawn, and plain radiographs of the chest and pelvis were obtained. The labs were significant for a leukocytosis of 34.98 × 10^3^ cells per cubic millimeter (cmm) (reference range: 4–11 × 10^3^/cmm). Chest and pelvis radiographs were unremarkable. Computed tomography of the head, cervical spine, chest, abdomen, and pelvis (Image 1 and 2) were subsequently performed.

Computed tomography revealed a rectus wall rupture with complete bowel herniation. After imaging, the patient was taken to the operating room for an exploratory laparotomy with small bowel resection, omentectomy, ileocecectomy, and reduction of the abdominal hernia. The surgical findings included a full-thickness small bowel injury, a bucket handle tear to the mesentery of the small bowel, a devitalized ischemic omentum, and a sigmoid colon serosal injury. Her abdomen was temporarily closed, and postoperative broad-spectrum antibiotics were initiated. The patient had five more abdominal surgeries, and after an 18-day hospital stay she was discharged to inpatient rehabilitation with an abdominal wound vacuum.

## DISCUSSION

This case describes a traumatic rectus abdominis muscle rupture with resulting large ventral hernia. This rare injury, sometimes referred to as a “handlebar hernia,” ^1^ is commonly associated with blunt abdominal trauma from a motorcycle handlebar.^1^ ^–^ ^2^ This case demonstrates the under-reported but more common mechanism for this traumatic abdominal wall hernia following an MVC.^3^ Three-point seatbelt harnesses are designed to secure over the sternum and hips, distributing deceleration forces in a way that decreases risk of serious injury. Improper use of a seatbelt, including use of the two-point lap belt only, can lead to significant injury^4^ including abdominal wall herniation. The force exerted on the abdomen by the lap belt leads to rupture of the anterior abdominal wall.

Computed tomography is the most sensitive diagnostic tool when evaluating for a rectus wall rupture. This patient’s abdominal wall rupture was classified as a grade V abdominal wall injury due to complete rectus abdominis muscle disruption with herniation of abdominal contents.^5^ Known risk factors for this condition include old age, weak abdominal muscles, and preexisting hernias. While rectus wall rupture with herniation is rare, it can be fatal if not treated immediately.^5^ The presence of abdominal wall ecchymosis, otherwise known as a “seatbelt sign,” after MVC is frequently associated with abdominal injuries.^5^

## References

[1] Vieira B Melo A Gomes C et al. Handlebar hernia: unusual complication from blunt trauma. J Surg Case Rep. 2022;2022(6):rjac195. 35673539 10.1093/jscr/rjac195 PMC9167937

[2] Chan KH Subramaniam S Haytai F. Traumatic abdominal wall hernia after impact from handlebar: a case report. Trauma Case Rep. 2021;36:100557. 34977317 10.1016/j.tcr.2021.100557 PMC8683998

[3] Netto F Hamilton P Rizoli SB et al. Traumatic abdominal wall hernia: epidemiology and clinical implications. J Trauma. 2006;61(5):1058–61. 17099509 10.1097/01.ta.0000240450.12424.59

[4] Herath M Bautz P Parker D et al. The importance of wearing a seatbelt correctly—a case report of blunt trauma causing complete shearing transection of the gastroduodenal junction. Int J Surg Case Rep. 2020;72:197–201. 32544828 10.1016/j.ijscr.2020.05.008 PMC7298554

[5] Pothiawala S Balasubramaniam S Taib M et al. Traumatic abdominal wall hernia: a rare and often missed diagnosis in blunt trauma. World J Emerg Med. 2022;13(6):492–4. 36636561 10.5847/wjem.j.1920-8642.2022.094 PMC9807386

